# Evaluating the Implementation and Clinical Effectiveness of an Innovative Digital First Care Model for Behavioral Health Using the RE-AIM Framework: Quantitative Evaluation

**DOI:** 10.2196/54528

**Published:** 2024-10-30

**Authors:** Samuel S Nordberg, Brittany A Jaso-Yim, Pratha Sah, Keke Schuler, Mara Eyllon, Mariesa Pennine, Georgia H Hoyler, J Ben Barnes, Lily Hong Murillo, Heather O'Dea, Laura Orth, Elizabeth Rogers, George Welch, Gabrielle Peloquin, Soo Jeong Youn

**Affiliations:** 1 Reliant Medical Group, OptumCare Worcester, MA United States; 2 United Health Group Minnetonka, MN United States; 3 University of Massachusetts Chan School of Medicine Worcester, MA United States; 4 Harvard Medical School Boston, MA United States

**Keywords:** digital mental health interventions, implementation, clinical effectiveness, practice-oriented research, access to care

## Abstract

**Background:**

In the United States, innovation is needed to address the increasing need for mental health care services and widen the patient-to-provider ratio. Despite the benefits of digital mental health interventions (DMHIs), they have not been effective in addressing patients’ behavioral health challenges as stand-alone treatments.

**Objective:**

This study evaluates the implementation and effectiveness of precision behavioral health (PBH), a digital-first behavioral health care model embedded within routine primary care that refers patients to an ecosystem of evidence-based DMHIs with strategically placed human support.

**Methods:**

Patient demographic information, triage visit outcomes, multidimensional patient-reported outcome measure, enrollment, and engagement with the DMHIs were analyzed using data from the electronic health record and vendor-reported data files. The RE-AIM (Reach, Effectiveness, Adoption, Implementation, Maintenance) framework was used to evaluate the implementation and clinical effectiveness outcomes of PBH.

**Results:**

PBH had a 47.58% reach rate, defined as patients accepting the PBH referral from their behavioral health integrated clinician. PBH patients had high DMHI registration rates (79.62%), high activation rates (76.54%), and high retention rates at 15 days (57.69%) and 30 days (44.58%) compared to literature benchmarks. In total, 74.01% (n=168) of patients showed clinical improvement, 22.47% (n=51) showed no clinical change, and 3.52% (n=8) showed clinical deterioration in symptoms. PBH had high adoption rates, with behavioral health integrated clinicians referring on average 4.35 (SD 0.46) patients to PBH per month and 90%-100% of clinicians (n=12) consistently referring at least 1 patient to PBH each month. A third (32%, n=1114) of patients were offered PBH as a treatment option during their triage visit.

**Conclusions:**

PBH as a care model with evidence-based DMHIs, human support for patients, and integration within routine settings offers a credible service to support patients with mild to moderate mental health challenges. This type of model has the potential to address real-life access to care problems faced by health care settings.

## Introduction

### Background

There is an increasing need for behavioral health services in the United States. It is estimated that 57.8 million, or 1 in 5 adults, in the United States experience mental health challenges [1], with some reports indicating challenges for 1 in 3 adults [2]. Despite this growing need for behavioral health services, it is well established that the United States will never be able to train enough behavioral health specialists to address the needs of the population adequately [3]. By 2024, the United States is expected to have a shortage of over 10,000 mental health care professionals [4] and will only have one provider available for every estimated 350 individuals with behavioral health needs [3]. Untreated behavioral health challenges have multi-level consequences. Mental health conditions have serious consequences for individuals’ health and are considered important risk factors for excess morbidity, including cardiovascular disease and diabetes [5,6], as well as premature mortality [7]. Further, individuals with untreated depression and anxiety are more likely to experience lost productivity in the workplace and produce lower-quality work [8]. Health care providers report increased burden and burnout as they address patients’ behavioral health needs without sufficient training, fewer opportunities for professional development, and facing competing demands between meeting productivity metrics and providing appropriate clinical care [9,10]. This gap in meeting behavioral health needs is also estimated to cost the United States over US $300 billion annually in overall lost productivity [11]. Thus, given the magnitude of consequences related to the access to care problem in behavioral health, it is imperative to find innovative and effective solutions.

### Digital Mental Health Interventions

Digital mental health interventions (DMHIs) have been proposed as a solution to mitigate the supply and demand gap. DMHIs are defined as “discrete digital functionalities of technology used to achieve health sector objectives, […] designed to achieve a specific outcome” [12], and can be delivered via mobile apps, web-based programs, wearables, and virtual reality headsets [13]. DMHIs are well poised to equip patients with needed behavioral health interventions and have the potential to reach more people for minimal additional cost per user relative to traditional modalities [14]. These tools are highly flexible and customizable, serving a wide variety of indications, from depression and anxiety to more serious mental illnesses such as bipolar disorder [14-16]. Further, they are more convenient for patients than traditional mental health care, removing scheduling and transportation barriers, thus allowing patients to use them where and when they want [17]. Lastly, DMHIs’ content is highly standardized and can, therefore, be delivered with high treatment fidelity [14].

Despite the significant benefits, stand-alone DMHIs have been shown to be insufficient in adequately addressing the demand for mental health services. The implementation of DMHIs has included significant end user adoption challenges, such as low registration, activation, and engagement or completion rates. For example, despite initial interest, only 42% of individuals visiting the website for a popular DMHI subsequently registered [15,18]. Even for those who do register, 70% of these users do not activate (ie, use the DMHI at least once post registration) [19], and low sustained engagement has been deemed the “Achilles heel” of DMHIs [20]. Baumel et al [21] reported that fewer than 4% of users who downloaded mental health apps engaged with the apps for at least 15-30 days. A systematic review of the uptake and use of digital self-help interventions identified that as few as 7% of those who registered with a DMHI had at least moderate engagement, and less than 1% completed the entire program [22].

A variety of barriers impede the adoption of DMHIs. Given there are between 10,000 and 20,000 DMHIs currently available [14,23,24], sorting through the options to select an appropriate DMHI may be challenging for users without input from a mental health clinician. Additionally, limited digital literacy has been shown to inhibit uptake and prolonged use [25,26]. Further, users seeking mental health treatment have expressed concerns about the efficacy of DMHIs [14,27] and indicated significantly greater interest in trying DMHIs that are explicitly recommended by clinicians and mental health care providers compared to those that are not [27]. Thus, there is an opportunity to address many of the current barriers to adopting DMHIs and maximize their benefits by embedding DMHIs within a clinical practice care model. Innovations that include the implementation of DMHIs in the clinic can efficiently increase access to timely and clinically appropriate care without the need for additional providers [28], as well as provide the resources patients need to successfully adopt them.

### Innovation: Precision Behavioral Health

Precision behavioral health (PBH) is a digital-first care model in which clinicians and staff support patients in the use of digital interventions drawn from an ecosystem of evidence-based DMHIs as a frontline treatment option. PBH and its implementation have been extensively described elsewhere [29]. Briefly, PBH aims to increase access to quality treatment for patients experiencing mild to moderate anxiety and depression symptoms. To do so, PBH was designed to maximize the benefits of DMHIs while simultaneously addressing the barriers to adoption, all within the context of a primary care-integrated behavioral health setting. The clinical work is wrapped in a practice-research network [30] that allows for its systematic evaluation.

There are several key components to the clinical deployment of PBH. First, PBH includes an ecosystem of DMHIs that have been pre-vetted to be evidence-based and clinically appropriate for the needs of the patients being served [29]. The DMHIs selected for the ecosystem address particular evidence-based mechanisms of change using well-established techniques such as mindfulness, biofeedback, diaphragmatic breathing, cognitive reappraisal, and others. Techniques are delivered through various mediums, including but not limited to virtual reality headsets, phone apps, and inhaler-style devices. Multiple mediums were purposely selected to have a variety of methods that could be matched with patient preferences and needs. The ecosystem of DMHIs has evolved as some DMHIs have been rejected and new ones have been added. At the time of this evaluation, 6 DMHIs were active in the ecosystem. The complete programming length for the DMHIs ranged from 4 to 12 weeks.

Second, PBH is embedded within a broader primary care-integrated behavioral health workflow. Here, patients identified by their primary care providers as needing behavioral health support are handed off to licensed behavioral health clinicians for an assessment and triage visit. During the triage visit, where clinically appropriate, the clinician can make a personalized referral to specific DMHIs that would be best suited for the patient’s needs. The triaging clinician also schedules a 4- or 6-week follow-up visit to check the patient’s progress and make new recommendations if necessary. Throughout the PBH program, patients’ symptoms are monitored at 2-week intervals via a computer adaptive outcomes questionnaire.

Lastly, PBH includes a strategically placed human connection explicitly designed to increase patients’ likelihood of registration and engagement with the DMHIs. After triage with a licensed clinician, patients meet with a non-clinical digital care navigator for technical support. This step has been shown to increase the patients’ registration rates from 20% to, on average, above 70% [31].

This paper aims to assess the clinical effectiveness and implementation outcomes of PBH. To evaluate whether an innovative care model such as PBH is a viable solution for providing and engaging patients with evidence-based digital mental health care, this paper uses the RE-AIM (Reach, Effectiveness, Adoption, Implementation, Maintenance) framework [32] to examine the reach, effectiveness, adoption, implementation, and maintenance of the PBH program.

## Methods

### Setting

Reliant Medical Group (Reliant) is a large multispecialty group medical practice in central Massachusetts, with an integrated behavioral health department available to provide consultation, triage, and treatment to patients in primary care. The behavioral health department includes a team of behavioral health integrated clinicians who serve as the first point of contact for patients identified by providers as needing integrated care. They receive warm handoffs from primary care providers, conduct crisis consultations, and conduct assessments to triage patients to appropriate behavioral health services. The behavioral health department at Reliant also includes a practice research network established in 2020 to improve patients’ access to high-quality care and efficiently generate effective and actionable outcomes to address research, clinical, and business needs [33]. The practice research network is embedded within the department and leverages practice-oriented data generated within the real-world practice setting to address clinically relevant problems through scientifically rigorous methods [30].

### Procedure

For a detailed description of the procedure for PBH, see [29]. Briefly, as part of routine clinical care at Reliant, patients referred from primary care for behavioral services meet with a behavioral health integrated clinician for an initial triage assessment. Before this meeting, patients are asked to complete a multidimensional patient-reported outcome measure (Norse Feedback [NF]) through the electronic health record patient portal. Behavioral health integrated clinicians are licensed mental health providers trained to conduct clinical evaluations to determine the course of treatment recommendation for the patients. During the initial triage assessment, the behavioral health integrated clinician meets with the patient to understand their behavioral health challenges and needs and uses their discussion, clinical judgment, the NF results, and other relevant clinical information to determine and subsequently refer patients to appropriate behavioral health treatment options, such as short-term goal-oriented therapy with Reliant providers, long-term therapy in the community, or PBH.

Patients who accept PBH are referred to a specific DMHI within the PBH ecosystem by the behavioral health integrated clinician based on the clinical information and other relevant patient-level characteristics, such as access to Wi-Fi, available privacy level, and patient preference. Patients who accept the DMHI referral are then sent registration information via the electronic health record and are immediately scheduled for a visit with the digital care navigator. The digital care navigator helps the patients register for the DMHI and address any other technical challenges experienced. Patients are outreached a total of 4 times post-referral as needed by the digital care navigator to aid their registration process. If a patient does not follow through with the registration after these outreaches, they receive a final message through the electronic health record portal encouraging them to reach out to their integrated clinician for follow-up.

**Figure 1:**
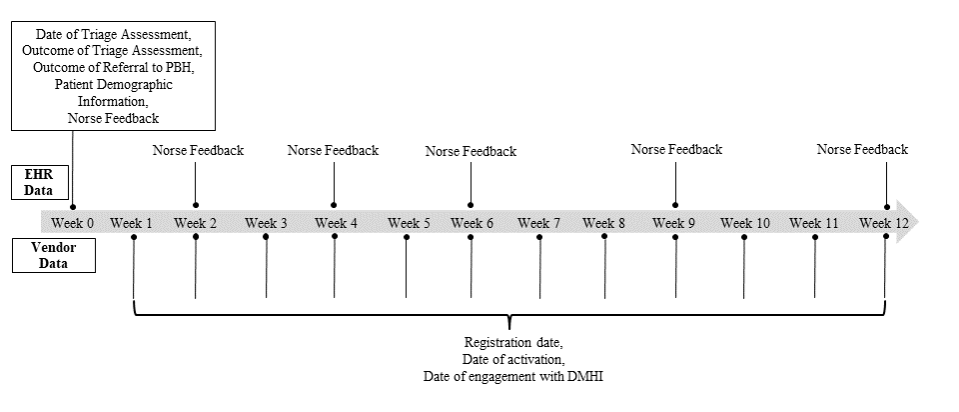
PBH measurement timeline for enrolled patients. Data were extracted from the EHR as well as from DMHI vendors from December 1, 2022 through July 31, 2023. PBH: precision behavioral health; EHR: electronic health data; DMHI: digital mental health intervention.

Patients then continue to complete the NF 2, 4, 6, 9, and 12 weeks post triage as part of the outcome monitoring component of PBH. The NF responses are closely monitored, and behavioral health integrated clinicians outreach patients who show clinically significant deterioration to evaluate further needs and discuss possible treatment changes. At 6 weeks, patients are also scheduled to meet with the behavioral health integrated clinician for a follow-up visit to inform any treatment decisions at that time. Figure 1 shows the NF assessment schedule.

### Ethical Considerations

The implementation and evaluation of PBH was determined to be an exempt quality improvement project by the Institutional Review Board at the Office of Human Research Affairs in United Health Group prior to data collection (Exemption action ID: 2023-0036-01). As part of routine care, referring providers collect verbal consent from patients referred to the PBH program to have the DMHI vendor share deidentified data using a randomly generated 9-digit ID regarding the patients’ enrollment, engagement, and clinical outcomes with Reliant. Given that PBH was implemented as part of routine care at Reliant, enrolled patients were not compensated for their participation. Patients were not financially responsible for access to the PBH program components or the DMHI program due to grant funding provided by United Health Group’s Strategy and Innovation Office to Reliant [29]. All results are shared at the group level to prevent any identification of individual patients.

### Data Collection

#### Electronic Medical Record System

PBH-related documentation was captured within the electronic medical record system as it was collected as part of standard documentation practices for the behavioral health integrated clinicians. The system recorded the date the behavioral health integrated clinicians met with patients to conduct the triage assessment, the outcome of the assessment (eg, PBH, psychiatry evaluation, goal-oriented therapy, group therapy, etc), and the outcome of referral to PBH (ie, patient accepted PBH referral or patient declined PBH referral). Patient demographic information and the NF responses were also collected and stored in the patient electronic medical record system.

#### NF

The NF measure [34,35] is a multidimensional patient-adapted self-report questionnaire. The NF was developed by conducting qualitative interviews with patients and clinicians to identify areas of clinical interest [34]. Following item development, the early version of the NF underwent evaluation in both clinical and nonclinical samples using item response theory, and items were removed or edited as necessary [34,35]. Each administration begins with “trigger items” or a single item representing an entire subscale; if patients score above a threshold on that trigger item, the remaining subscale questions are offered later during the assessment for patient completion. Once the subscale has been activated in an assessment, it remains open, in that all the subscale items are shown to the patient for completion (regardless of trigger score item score) until the average of that subscale for the subsequent 3 NF administrations is below a subclinical closing threshold. Due to the dynamic nature of the questionnaire, the items in the NF can range from 27 to 96 questions. Total scores are not calculated for the NF; instead, each subscale has its own score.

The NF measure includes 22 different subscales covering various domains, such as psychopathology symptoms, chronic maintaining factors, consequences of mental illness, and personal resources. These 22 subscales are anger, cognitive problems, general functioning, hopelessness, internal avoidance, pain, physical anxiety, readiness for change, eating concerns, sad affect, social support, substance use, self-compassion, self-contempt, social avoidance, traumatic memories, urges, worry, and a few single-item questions that address sexuality/sex functioning, physical health, sleep, and self-harm. NF question responses are recorded on a 7-point Likert scale ranging from 1, “*This is not at all true for me*,” to 7, “*This is completely true for me*,” for all questions included in the analysis. Any subscale whose subscale total score is higher than the trigger score is identified as “clinically elevated” and highlighted in the patient's medical chart for clinical review and appropriate response as needed.

#### DMHI Vendor Data

The business relationship established between PBH and each DMHI vendor included systematic data collection and sharing from the vendors with PBH. The variables sent by each vendor differed as the data sent were responsive to each DMHI’s unique programming. However, PBH asked each DMHI vendor to send standardized variables to evaluate the effectiveness of the PBH care model as a whole. Specifically, all DMHI vendors sent PBH data related to the patient’s registration date and date of activation, which was defined as the date when the patient engaged with the DMHI content for the first time post registration (this could be the same day, or this could be days after registration, or this variable would be missing for patients that registered but never engaged with the DMHI further), as well as the date of engagement with DMHI. We calculated engagement with the DMHI as the number of interactions the patient had with the DMHI per week. Initially, vendors shared data with PBH on weekly cadences, which changed into daily data transfers. Data were shared using a secure web-based file transfer application.

### Data Selection

PBH was first integrated within routine clinical care at Reliant on April 25, 2022. The preimplementation phase [36] lasted until December 1, 2022. It focused on customizing the delivery components of PBH, including the gradual rollout of the various DMHIs in the ecosystem; the inclusion of the NF as the main outcome monitoring measure within the behavioral health department in October 2022; the development and refinement of the operational workflows related to the integration of PBH into routine clinical care; the implementation of extensive DMHIs and PBH-related training for the behavioral health–integrated clinicians; and the establishment of the infrastructure needed for data capture, ensuring data quality, and workflow support within the electronic health record system. Thus, the data pertaining to the evaluation of PBH’s implementation outcomes and effectiveness include patients with a baseline and posttreatment assessment completed from December 1, 2022, through July 31, 2023 (date data pulled for this evaluation), during the implementation phase [36].

Demographic information for patients enrolled until November 30, 2022, is included in Table 1, and for patients enrolled after December 1, 2022, their demographic characteristics are summarized in Table 2. To evaluate whether there were meaningful differences in the patients enrolled during the preimplementation and implementation phases, we conducted chi-square tests, Fisher exact test, and the Welch 2-sample *t*-test. The results showed that there were no significant differences in the 2 samples’ legal sex (*χ*^2^_2_[N=1024]=2.4, *P*=.30) race (*P*=.20), and ethnicity (*χ*^2^_2_[N=1024]=1.5, *P*=.50). Significant differences were observed for age (t_1019_=2.28, *P*=.02), which implies that among the groups included in our analyses, patients who were offered and accepted the PBH referral after December 1, 2022, were older (mean 41.59, SD 16.04 years) than those who were offered and accepted the referral before November 30, 2022 (mean 37.88, SD 14.42 years).

**Table 1:** Demographics of patients (N=494) who accepted the PBH^a^ referral from April 25, 2022, through November 30, 2022.

Variable		Values
Age (years), mean (SD)		37.88 (14.42)
**Legal sex,** **n** **(%)**		
	Female	364 (73.68)
	Male	128 (25.91)
	N/A^b^	2 (0.40)
**Race,** **n** **(%)**		
	White	332 (67.21)
	Black or African American	17 (3.44)
	Asian	3 (0.61)
	Biracial	14 (2.83)
	Native American	2 (0.40)
	Multiracial	1 (0.20)
	Pacific Islander	0 (0)
	N/A	125 (25.30)
**Ethnicity,** **n** **(%)**		
	Not Hispanic or Latino	283 (57.29)
	Hispanic or Latino	53 (10.73)
	Unknown	158 (31.98)

^a^PBH: precision behavioral health.

^b^N/A: not available.

**Table 2:** Demographics of patients who attended a triage visit through the different phases of PBH enrollment from December 1, 2022, through July 31, 2023.

		Triage visit	PBH offered^a^	PBH declined^b^	PBH accepted^b^	DMHI registered^c^	DMHI activated^d^
Total, n		3457	1114	584	530	422	323
Age (years), mean (SD)		41.59 (16.04)	40.23 (15.10)	40.45 (15.11)	39.99 (15.11)	39.88 (15.36)	40.33 (15.49)
**Legal sex,** **n** **(%)**							
	Female	2310 (66.82)	798 (71.63)	399 (68.32)	399 (75.28)	322 (76.30)	252 (78.02)
	Male	1144 (33.09)	316 (28.37)	185 (31.68)	131 (24.72)	100 (23.70)	71 (22.18)
	Nonbinary	3 (0.09)	0 (0)	0 (0)	0 (0)	0 (0)	0 (0)
**Race,** **n** **(%)**							
	White	2470 (71.45)	799 (71.72)	427 (73.12)	372 (70.19)	303 (71.80)	234 (72.45)
	Black or African American	131 (3.79)	52 (4.67)	24 (4.11)	28 (5.28)	24 (5.69)	17 (5.26)
	Asian	60 (1.74)	17 (1.53)	10 (1.71)	7 (1.32)	4 (0.95)	3 (0.93)
	Biracial	54 (1.56)	17 (1.53)	9 (1.54)	8 (1.51)	5 (1.18)	3 (0.93)
	Native American	16 (0.46)	5 (0.45)	2 (0.34)	3 (0.57)	3 (0.71)	2 (0.62)
	Multiracial	7 (0.20)	2 (0.18)	1 (0.17)	1 (0.19)	1 (0.24)	1 (0.31)
	Pacific Islander	4 (0.12)	0 (0)	0 (0)	0 (0)	0 (0)	0 (0)
	N/A^e^	715 (20.69)	222 (19.93)	111 (19.01)	111 (20.94)	82 (19.43)	63 (19.51)
**Ethnicity,** **n** **(%)**							
	Non-Hispanic or Latino	2090 (60.46)	686 (61.58)	367 (62.84)	319 (60.19)	259 (61.37)	194 (60.06)
	Hispanic or Latino	340 (9.84)	118 (10.59)	58 (9.93)	60 (11.32)	42 (9.95)	34 (10.53)
	N/A	1027 (29.71)	310 (27.83)	159 (27.23)	151 (28.49)	121 (28.68)	95 (29.41)

^a^PBH (precision behavioral health) offered: integrated clinicians believed the patients to be eligible for PBH and offered to refer the patients to the program.

^b^PBH declined/accepted: count of the patients’ decision whether to accept or decline the PBH referral from the integrated clinician.

^c^DMHI (digital mental health intervention) registered: patients who were deemed eligible, offered PBH, accepted the referral and signed up using the DMHI’s specific registration procedures.

^d^DMHI activated: patients who were deemed eligible, offered PBH, accepted the referral, signed up using the DMHI’s specific registration procedures, and engaged with their DMHI at least once.

^e^N/A: not available.

### Data Analysis

To evaluate the outcomes of PBH, we used the RE-AIM framework [32] to assess the relevant implementation and effectiveness outcomes [29].

*Reach* was determined as the proportion of patients that accepted the referral made by the behavioral health integrated clinician. To further characterize the reach patients for PBH in the context of all patients being referred for behavioral health services, we summarized and compared the demographic characteristics of patients who attended a behavioral health triage assessment visit, patients who were referred to PBH, patients who accepted the PBH referral, and patients who were identified as eligible for PBH by their behavioral health integrated clinician but the patient decided against participation.

The *effectiveness* of the PBH program was assessed in two ways. First, we analyzed the patients’ behaviors as they related to the DMHIs. We summarized the patients’ DMHI registration rates post referral, their activation rates (defined as patients who engaged at least once with the DMHI post-registration [19]), and retention rates at 15 and 30 days (defined as the percentage of patients who engaged with the DMHI at least once weekly out of the number of activated patients). Second, we evaluated patient-level clinical outcomes of interest. PBH’s clinical response rate was analyzed as the number of patients classified as having clinically improved, clinically worsened, or shown no clinical improvement in the NF measure [34]. To assess clinical change, we calculated the amount of change observed between the baseline and 6-week follow-up NF assessments for each of the NF subscales that were clinically elevated during baseline. The 6-week follow-up NF assessment, or the closest NF assessment to this time frame after 6 weeks, was selected as it was reflective of the time for most patients to have had access to half of the program for most of the digital interventions in the ecosystem. Following the definition by Hiller and colleagues [37], for a patient to be categorized as having clinically improved, they had to have achieved more than 50% improvement from baseline to 6-week follow-up NF scores in the pathological range, the follow-up NF score had to be at least 25% lower than the baseline score, and these criteria had to be met on at least 25% of the baseline elevated NF subscales. For a patient to be categorized as having clinically deteriorated, they had to show more than 50% symptom deterioration on the NF scores from baseline to 6-week follow-up in the pathological range, the follow-up NF score had to be at least 25% higher than the baseline score, and these criteria had to be met on at least 25% of the baseline elevated NF subscales. If a patient was not categorized as either clinically improved or clinically worsened, they were categorized as showing no clinical change. Detailed information on the categorization development can be found in Multimedia Appendix 1.

Given that PBH was implemented as part of routine care within the behavioral health department at Reliant, all behavioral health integrated clinicians *adopted* PBH [29]. Behavioral health integrated clinicians met biweekly to discuss PBH, and the DMHIs within the ecosystem, and share patient cases that were referred versus not referred to PBH to support the continued adoption of PBH. To further characterize the adoption behaviors of the providers, we tracked the average number of referrals made by the behavioral health integrated clinicians to PBH each month, as well as the percent of clinicians who made 1, 2, and 3 patient referrals to PBH per month among the 10-12 active clinicians.

*Implementation* was evaluated at the behavioral health integrated clinician level. We assessed the total number of patients referred to PBH out of those who attended a triage assessment visit.

*Maintenance* was defined as the extent to which PBH became a part of routine practice at Reliant, in addition to any long-term effects of PBH after it concluded [32]. Since PBH is still in its implementation phase [36] as part of clinical care at Reliant, we are unable to evaluate the maintenance of PBH at this time.

## Results

### Reach

The proportion of patients reached by the PBH program was 47.58% (530 patients who accepted the PBH referral/1114 patients referred to PBH by an integrated clinician). Table 2 shows the demographic characteristics of patients who attended a behavioral health triage assessment, patients who were referred to PBH, patients who accepted the PBH referral, patients who declined the PBH referral, patients who successfully registered, and patients who activated their DMHI. Chi-square tests and a 1-way ANOVA were used to assess significant demographic differences between the patient groups in Table 2. Our results indicate significant differences in legal sex (*χ*^2^_5_[N=6427]=42.6, *P*<.01). It should be noted that nonbinary sex was excluded from these results due to its absence in all groups except for patients who attended a triage visit. Logistic regression with estimated marginal means was used to further investigate the differences between the groups for legal sex. Results indicate that the female-to-male ratio of patients offered PBH was significantly higher (odds ratio 1.25, 95% CI 1.01-1.55) than the female-to-male ratio at triage, indicating that females were offered PBH more frequently than males. No additional significant differences between groups were found. Results were nonsignificant for ethnicity (*χ*^2^_10_[N=6430]=3.9, *P*=.90) and race (*χ*^2^_35_[N=6430]=16.4, *P*=.99). One-way ANOVA was significant for age (*F*_5,6424_=3206, *P*<.05). Post hoc Bonferroni corrections did not yield any significant differences between the groups, likely due to a lack of sensitivity compared to the 1-way ANOVA.

### Effectiveness

In terms of assessing PBH patients’ behaviors with their referred DMHIs, among patients referred to PBH who accepted the referral (n=530), 79.62% (n=422) of patients who accepted the PBH referral continued on to register with their DMHI. Of those registered patients, 76.54% (n=323) activated their DMHI. Patient retention rates at 2 and 4 weeks were 57.69% and 44.58%, respectively.

For the clinical change analysis, 9 (2.79%) DMHI-activated patients did not have any clinical elevations in any of the NF subscales at baseline, and, thus, were not included. Additionally, 87 (26.93%) patients were excluded from the analysis because they lacked posttreatment assessment data. Demographic descriptions and the Welch *t* test and chi-square comparisons between these excluded patients and the included patients are found in Multimedia Appendix 2. The results show no significant differences in age (t_130_=0.44, *P*=.66); legal sex (*χ*^2^_1_[N=410]=0.1, *P*=.78); race (*P*=.11); or ethnicity (*χ*^2^_2_[N=410]=0.4, *P*=.83) between the 2 groups. More than two-thirds of the remaining 227 patients with elevated NF baseline scores showed clinical improvement (n=168, 74.01%), 22.47% (n=51) of the patients showed no change in clinical symptoms, and 3.52% (n=8) of the patients showed deterioration in clinical symptoms.

### Adoption

**Figure 2:**
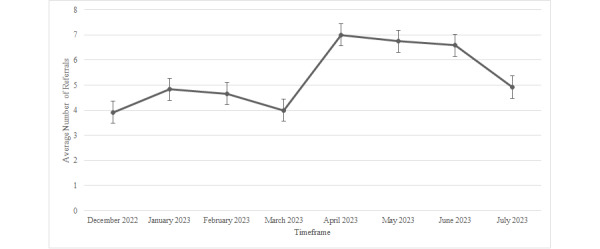
Average number of PBH referrals made by behavioral health integrated clinicians per month from December 1, 2022, through July 31, 2023a. PBH: precision behavioral health; aError bars: standard error.

**Figure 3:**
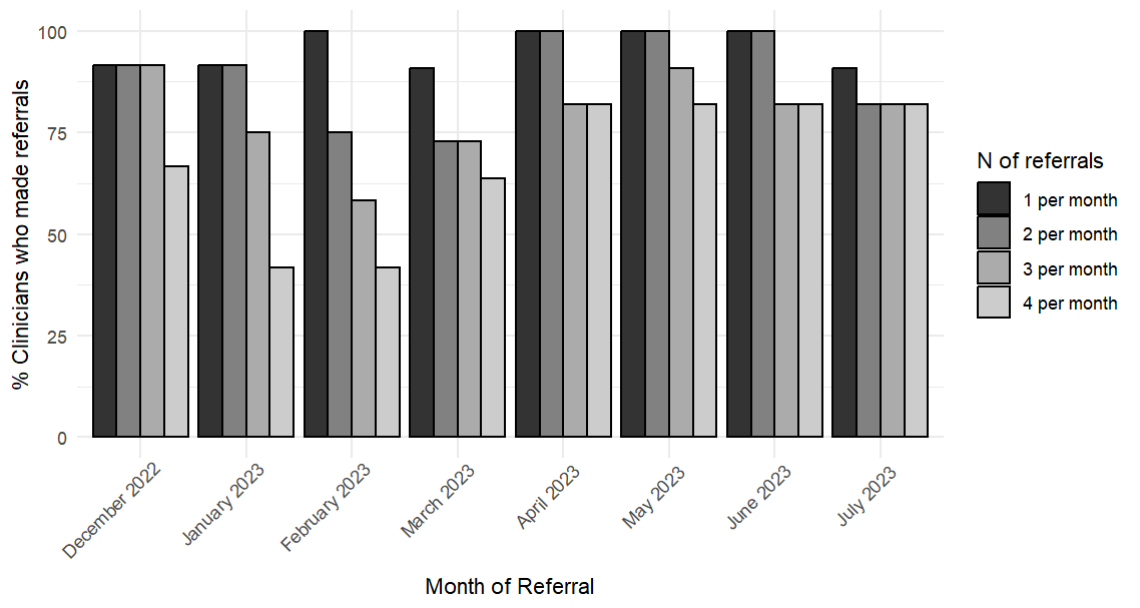
Bar graph representing the percent of integrated clinicians who made 1, 2, and 3 referrals to PBH each month from December 1, 2022, through July 31, 2023. PBH: precision behavioral health.

The average number of referrals made by behavioral health integrated clinicians to PBH each month over the course of the analytic period is illustrated in Figure 2. On average, the integrated clinicians made 5.33 (SD 1.17) PBH referrals per month across the study period. There was an average of 4.35 (SD 0.46) PBH referrals per month per clinician from December 2022 to March 2023. The average number of referrals per month increased to 6.78 (SD 0.21) per clinician between April 2023 and June 2023. In July 2023, there was a slight decrease in the average number of referrals per month to 4.92 (SD 3.60). Because the lowest mean number of referrals was three throughout the study period, Figure 3 shows the percentage of clinicians (n=12) who made 1, 2, and 3 referrals each month. The percentage of clinicians who made at least 1 referral for any given month ranged from 90% to 100%; 72% to 100% for 2 referrals each month; and 58%-91% for 3 referrals.

### Implementation

During the analytic period, a total of 3457 patients attended a behavioral health triage visit, and 1114 (32%) were offered PBH (Table 2).

## Discussion

To adequately address the access to care problem in behavioral health, we need innovative and scalable solutions that will have a meaningful impact on patients and health care organizations. Thus, this paper aimed to evaluate the implementation and effectiveness outcomes of PBH, a digital-first behavioral health care model that was integrated as part of routine clinical care, with an ecosystem of evidence-based DMHIs as treatment options and strategically placed human support for patients. Given the significant differences between naturalistic studies and randomized controlled trials in both engagement and effectiveness of DMHIs [38,39], the focus of our study was to assess the effectiveness of PBH as delivered in routine care to adequately understand its real-world impact and applications.

The implementation outcomes results show that almost 50% of patients were reached, defined as patients accepting the referral to PBH as a behavioral health treatment option. Despite some research showing that patients may be hesitant to accept digital tools as valid mental health options [26], our results provide support for embedding DMHIs within a care model, with provider referral possibly addressing patient-level barriers and increasing reach rates [27]. There were significantly more females referred to PBH compared to males. This could reflect the fact that females are more represented in behavioral health patient populations [40] or that there was a provider bias present in the patients that were referred to PBH. Provider adoption rates were high. The results show that the behavioral health integrated clinicians, on average, referred more than 4 patients per month and that a high percentage of clinicians referred 2-3 patients in a given month. The current model of having providers adopt innovation in the context of routine clinical care and a practice research network shows that practice-oriented research [30] is a complementary paradigm to implementation efforts in increasing provider adoption rates [41]. The implementation outcomes further support the adoption results. The behavioral health integrated clinicians conducting the triage visit offered PBH to 1 out of 3 patients attending these visits as an alternative treatment option. These results show that innovative clinical care models such as PBH can bridge the gap in mental health care by expanding treatment pathways to include evidence-based DMHIs. Patients getting diverted from traditional psychotherapy referrals and into these types of digital interventions means that they will access treatment right away while freeing up therapy resources for patients that may be better suited for traditional treatment modalities.

The results of the clinical effectiveness evaluation further support PBH as an effective treatment option for patients. More than 70% of PBH patients showed clinical-level improvement rates, as compared to the 38% clinical improvement rates found in psychotherapy delivered in routine clinical care settings [42]. It should be noted that the 3.5% of PBH patients that showed clinical-level deterioration of symptoms is lower than the 5% deterioration rates found in routine psychotherapy [40]. However, given the small sample size of deteriorating patients in the program (n=8), these results should be interpreted with caution. PBH patients’ usage of the DMHIs was also high; almost 80% of PBH patients registered with their DMHI, compared to 42% reported in previous studies [15,18], 76% activated their DMHI post registration, which is higher than the 30% found in the literature [19], and patient retention rates were almost 58% and 45% at 15 days and 30 days, compared to 4% and 3%, respectively, as assessed by the systematic review by Baumel et al [21]. The PBH program’s inclusion of a digital care navigator, deployed to assist patients with their DMHIs post-referral from a clinician, has shown to increase registration success rates significantly [31]. Additionally, we hypothesize that the follow-up check-in visit with the behavioral health integrated clinician may aid PBH’s high retention rates. Patients may feel a sense of accountability as well as support throughout their DMHI journey since they have a dedicated, scheduled visit with their referring provider to review treatment progress or discuss alternative treatment options.

The results of the study should also be considered in the context of a few limitations. About 27% of patients who had activated their DMHIs were not included in the effectiveness analysis because of a lack of post-outcome measure completion. This missing rate is comparable to other data collected in naturalistic settings [43], and there were no differences between patients who registered compared to those that activated. Nonetheless, the effectiveness results reported should be interpreted with this limitation. Additionally, this study focused on overall clinical improvement, regardless of DMHI usage. There is evidence to suggest that differential usage patterns of DMHIs lead to different outcomes in depression and anxiety, with some studies showing that more engagement is related to improved outcomes [44], whereas others not finding a relationship between engagement and effectiveness [45]. Thus, future studies should examine whether there are patterns of DMHI usage within the PBH ecosystem and how they relate to clinical improvement. Additionally, how DMHI usage is defined has varied in the field; some focused on daily usage, whereas others on weekly [46]. This variability is expected as DMHIs have different program components and suggested frequency of engagement. This study standardized these definitions within the DMHIs in the PBH ecosystem to define engagement as at least once a week engagement, which is consistent with how it has been done in the field. Future studies should explore the impact that differing ways of defining engagement have on overall metrics as well as outcomes. Lastly, this study did not evaluate patient-level characteristics that may have impacted them agreeing or declining PBH or registering with the DMHI post-referral. It would be beneficial for future investigations to focus on understanding what are patient-level factors that impact their decision to accept and start engaging with DMHIs.

Overall, the implementation and effectiveness results for PBH provide evidence for implementing these kinds of solutions in actual care delivery organizations. Chief medical officers or other leadership staff of health care settings would be equipped with the information they need to evaluate whether PBH and related programs could address the behavioral health access to care problems they are challenged with day in and day out. PBH’s reach, effectiveness, adoption, and implementation outcome results show that PBH can and should be considered as a referral option for patients in addition to traditional psychotherapy options; the high uptake, retention rates, and effectiveness equivalence to psychotherapy as delivered in routine care provide leadership the metrics that they need to reconsider their existing treatment services and confidently refer patients to innovative and impactful referral options, such as PBH.

## Appendix

Multimedia Appendix 1 Additional materials.

Multimedia Appendix 2 Additional materials 2.

## Abbreviations

DMHI: digital mental health intervention
NF: Norse Feedback
PBH: precision behavioral health
RE-AIM: Reach, Effectiveness, Adoption, Implementation, Maintenance

## Data Availability

The data analyzed during this study are not publicly available as data were collected as part of routine care provision at Reliant.

## References

[1] Mental illness. National Institute of Mental Health.

[2] Panchal N, Saunders H, Rudoqitz R, Cox C Implications of COVID-19 for mental health and substance use. KFF.

[3] Reinert M, Fritze D, Nguyen T (2022). The State of Mental Health in America 2022.

[4] U.S. Department of Health and Human Services, Health Resources and Services Administration, Bureau of Health Workforce, National Center for Health Workforce Analysis (2016). National Projections of Supply and Demand for Behavioral Health Practitioners: 2013-2025.

[5] Rotella F, Mannucci E (2013). Depression as a risk factor for diabetes: a meta-analysis of longitudinal studies. J Clin Psychiatry.

[6] Sowden GL, Huffman JC (2009). The impact of mental illness on cardiac outcomes: a review for the cardiologist. Int J Cardiol.

[7] Druss BG, Zhao L, Von Esenwein S, Morrato EH, Marcus SC (2011). Understanding excess mortality in persons with mental illness: 17-year follow up of a nationally representative US survey. Med Care.

[8] Sanderson K, Andrews G (2006). Common mental disorders in the workforce: recent findings from descriptive and social epidemiology. Can J Psychiatry.

[9] Substance Abuse and Mental Health Services Administration (SAMHSA) (2022). Substance Abuse and Mental Health Services Administration.

[10] Rollins AL, Eliacin J, Russ-Jara AL, Monroe-Devita M, Wasmuth S, Flanagan ME, Morse GA, Leiter M, Salyers MP (2021). Organizational conditions that influence work engagement and burnout: a qualitative study of mental health workers. Psychiatr Rehabil J.

[11] FY 2018 funding for mental health. National Alliance on Mental Illness.

[12] Substance Abuse and Mental Health Services Administration (2023). Digital Therapeutics for Management and Treatment in Behavioral Health. Publication No. PEP23- 06-00-001.

[13] Khanna M, Rose R (2022). Digital interventions in mental health. Cogn Behav Pract.

[14] Schueller SM, Torous J (2020). Scaling evidence-based treatments through digital mental health. Am Psychol.

[15] Fleming T, Bavin L, Lucassen M, Stasiak K, Hopkins S, Merry S (2018). Beyond the trial: systematic review of real-world uptake and engagement with digital self-help interventions for depression, low mood, or anxiety. J Med Internet Res.

[16] Nicholas J, Larsen ME, Proudfoot J, Christensen H (2015). Mobile apps for bipolar disorder: a systematic review of features and content quality. J Med Internet Res.

[17] Schueller SM, Hunter JF, Figueroa C, Aguilera A (2019). Use of digital mental health for marginalized and underserved populations. Curr Treat Options Psych.

[18] Batterham PJ, Neil AL, Bennett K, Griffiths KM, Christensen H (2008). Predictors of adherence among community users of a cognitive behavior therapy website. Patient Prefer Adherence.

[19] Agachi E, Bijmolt THA, van Ittersum K, Mierau JO (2023). The effect of periodic email prompts on participant engagement with a behavior change mHealth app: longitudinal study. JMIR Mhealth Uhealth.

[20] Nwosu A, Boardman S, Husain MM, Doraiswamy PM (2022). Digital therapeutics for mental health: is attrition the achilles heel?. Front Psychiatry.

[21] Baumel A, Muench F, Edan S, Kane JM (2019). Objective user engagement with mental health apps: systematic search and panel-based usage analysis. J Med Internet Res.

[22] Christensen H, Griffiths KM, Korten AE, Brittliffe K, Groves C (2004). A comparison of changes in anxiety and depression symptoms of spontaneous users and trial participants of a cognitive behavior therapy website. J Med Internet Res.

[23] Clay RA Mental health apps are gaining traction: American Psychological Association 2021 annual trends report.

[24] Torous J, Roberts LW (2017). Needed innovation in digital health and smartphone applications for mental health: transparency and trust. JAMA Psychiatry.

[25] Nouri SS, Avila-Garcia P, Cemballi AG, Sarkar U, Aguilera A, Lyles CR (2019). Assessing mobile phone digital literacy and engagement in user-centered design in a diverse, safety-net population: mixed methods study. JMIR Mhealth Uhealth.

[26] Borghouts J, Eikey E, Mark G, De Leon C, Schueller SM, Schneider M, Stadnick N, Zheng K, Mukamel D, Sorkin DH (2021). Barriers to and facilitators of user engagement with digital mental health interventions: systematic review. J Med Internet Res.

[27] Lipschitz J, Miller CJ, Hogan TP, Burdick KE, Lippin-Foster R, Simon SR, Burgess J (2019). Adoption of mobile apps for depression and anxiety: cross-sectional survey study on patient interest and barriers to engagement. JMIR Ment Health.

[28] Webb TL, Joseph J, Yardley L, Michie S (2010). Using the internet to promote health behavior change: a systematic review and meta-analysis of the impact of theoretical basis, use of behavior change techniques, and mode of delivery on efficacy. J Med Internet Res.

[29] Youn S, Jaso B, Eyllon M, Sah P, Hoyler G, Barnes JB, Jarama K, Murillo L, O'Dea H, Orth L, Pennine M, Rogers E, Welch G, Nordberg SS (2024). Leveraging implementation science to integrate digital mental health interventions as part of routine care in a practice research network. Adm Policy Ment Health.

[30] Castonguay LG, Barkham M, Youn S, Page AC (2021). Practice-based evidence—findings from routine clinical settings. Bergin and Garfield's Handbook of Psychotherapy and Behavior Change: 50th Anniversary Edition, 7th ed.

[31] Jaso-Yim Brittany, Eyllon Mara, Sah Pratha, Pennine Mariesa, Welch George, Schuler Keke, Orth Laura, O'Dea Heather, Rogers Elizabeth, Murillo Lily H, Barnes J Ben, Hoyler Georgia, Peloquin Gabrielle, Jarama Kevin, Nordberg Samuel S, Youn Soo Jeong (2024). Evaluation of the impact of a digital care navigator on increasing patient registration with digital mental health interventions in routine care. Internet Interv.

[32] What is RE-AIM?.

[33] Barnes BJ (2023). Building a practice research network within an integrated BH department of a large medical practice.

[34] McAleavey AA, Nordberg SS, Moltu C (2021). Initial quantitative development of the norse feedback system: a novel clinical feedback system for routine mental healthcare. Qual Life Res.

[35] Nordberg SS, McAleavey AA, Moltu C (2021). Continuous quality improvement in measure development: lessons from building a novel clinical feedback system. Qual Life Res.

[36] Kilbourne AM, Neumann MS, Pincus HA, Bauer MS, Stall R (2007). Implementing evidence-based interventions in health care: application of the replicating effective programs framework. Implement Sci.

[37] Hiller W, Schindler AC, Lambert MJ (2012). Defining response and remission in psychotherapy research: a comparison of the RCI and the method of percent improvement. Psychother Res.

[38] Granek B, Evans A, Petit J, James MC, Ma Y, Loper M, Fuccillo M, Schmidt R (2021). Feasibility of implementing a behavioral economics mobile health platform for individuals with behavioral health conditions. Health Technol.

[39] Meyerowitz-Katz G, Ravi S, Arnolda L, Feng X, Maberly G, Astell-Burt T (2020). Rates of attrition and dropout in app-based interventions for chronic disease: systematic review and meta-analysis. J Med Internet Res.

[40] Terlizzi EP, Zablotsky B (2019). Mental Health Treatment Among Adults: United States.

[41] Youn S, Boswell JF, Douglas S, Harris BA, Aajmain S, Arnold KT, Creed TA, Gutner CA, Orengo-Aguayo R, Oswald JM, Stirman SW (2024). Implementation science and practice-oriented research: convergence and complementarity. Adm Policy Ment Health.

[42] Cuijpers P, Karyotaki E, Ciharova M, Miguel C, Noma H, Furukawa TA (2021). The effects of psychotherapies for depression on response, remission, reliable change, and deterioration: a meta-analysis. Acta Psychiatr Scand.

[43] Asnaani A, Benhamou K, Kaczkurkin AN, Turk-Karan E, Foa EB (2020). Beyond the constraints of an RCT: naturalistic treatment outcomes for anxiety-related disorders. Behav Ther.

[44] Gan DZQ, McGillivray L, Han J, Christensen H, Torok M (2021). Effect of engagement with digital interventions on mental health outcomes: a systematic review and meta-analysis. Front Digit Health.

[45] Chien I, Enrique A, Palacios J, Regan T, Keegan D, Carter D, Tschiatschek S, Nori A, Thieme A, Richards D, Doherty G, Belgrave D (2020). A machine learning approach to understanding patterns of engagement with internet-delivered mental health interventions. JAMA Netw Open.

[46] Lipschitz JM, Van Boxtel R, Torous J, Firth J, Lebovitz JG, Burdick KE, Hogan TP (2022). Digital mental health interventions for depression: scoping review of user engagement. J Med Internet Res.

