# Supplementary material for: Evaluating the Implementation and Clinical Effectiveness of an Innovative Digital First Care Model for Behavioral Health Using the RE-AIM Framework: Quantitative Evaluation
Source: J Med Internet Res. 2024 Oct 30;26:e54528. doi: 10.2196/54528 (PMC11561446; doi:10.2196/54528)
Supplement: Multimedia Appendix 1 [file jmir_v26i1e54528_app1.docx]

# Supplemental Material 1.

Treatment response to Precision Behavioral Health was calculated based on the Percent of Improvement Approach (PI) as defined by Hiller et al. (2011). The PI approach includes calculating the percentage of improvement in patient outcomes from pre- to post-treatment, taking into account symptom severity at baseline. A significant benefit of using the PI approach method is that patient improvement or deterioration is expressed as a percentage. This allows for easy comparison across different individuals and studies, facilitating a standardized evaluation of treatment effectiveness.

Hiller et al. (2011) defined two criteria for patient improvement:

1. At least 50% improvement from baseline score to treatment end. The pathological range is considered while computing the degree of change for an individual patient; and
2. At least 25% improvement on the entire range of the instrument used for measuring patient outcome.

We describe below the steps taken to apply the Hiller et al. (2011) criteria to evaluate clinical improvement in patients referred to PBH using the Norse Feedback (NF; McAleavey et al., 2021; Nordberg et al., 2021) measure, a multidimensional patient-adapted self-report outcome measure. The NF is described in detail in the body of the manuscript. Briefly, it includes 22 subscales (Table 1), that cover psychopathology symptoms, chronic maintaining factors, consequences of mental illness, and personal resources. Higher scores on the NF are related to more severe symptoms. Each administration begins with a “trigger item”, or a single item that represents that subscale. If a patient’s response to the trigger item is above the predetermined threshold, the remaining subscale questions are offered to the patient for completion and the subscale is considered to be clinically elevated; if the response is below the threshold, the patient is not asked additional questions within that subscale. The NF does not have a total score; instead, each subscale has its own subscale score.

Table 1: Norse Feedback subscales

| Norse Feedback subscales name |
| --- |
| Anger |
| Physical Anxiety |
| Readiness For Change |
| Cognitive Problems |
| Self Compassion |
| Self Contempt |
| Eating Concerns |
| General Functioning |
| Physical Health |
| Hopelessness |
| Internal Avoidance |
| Traumatic Memories |
| Pain |
| Sad Affect |
| Social Avoidance |
| Sexuality/ Sex Life |
| Self Harm |
| Sleep |
| Substance Use |
| Social Support |
| Urges |
| Worry |

# Procedures

For patients that were referred to and accepted the PBH referral, we considered their pre-treatment (baseline) scores to be the NF measure collected within a week of their triage assessment. The follow-up score was the NF completed at 6-weeks post triage assessment (or if absent, any NF score collected closest to the 6-weeks post-triage assessment visit). Changes in subscale score for each of the 22 NF subscales were computed by subtracting the pre-treatment score from the follow-up score. For a given patient, the change in subscale score was only evaluated for the NF subscales that were elevated at baseline (i.e., with a subscale score exceeding the scale-specific opening threshold score).

### Clinically significant improvement definition

Following the two criteria postulated by Hiller at al. (2011), a patient was categorized as having shown clinically significant improvemrestent at follow-up if the following criteria were met:

Criteria 1: For NF subscales that were clinically elevated at baseline, the follow-up NF score showed at least 50% improvement in the pathological range. The pathological range was defined as the range between the NF subscale specific opening threshold and the baseline score. For example, for a NF subscale with an opening threshold of 3, a patient with baseline score of 6 and follow-up score of 4 represents a reduction of 66.67% (2 points actual improvement relative to 3 points of possible improvement between 6 and 4) and would be considered as improved for that particular NF subscale.

Criteria 2**:** For NF subscales that were clinically elevated at baseline, the follow-up score should be at least 25% lower than the baseline score;

Criteria 3: Since the Hiller et al. (2011) definition was developed for unidimensional measures, and the NF is a multidimensional measure, we added a 3^rd^ criterion to determine whether a patient had shown clinical improvement (detailed development description can be found later in this supplementary material). Specifically, a patient was categorized as “clinically improved” if at least 25% of the patient's NF subscales that had been elevated at baseline showed clinical improvement (i.e., met criteria 1 and 2).

### Clinically significant worsening definition

We followed a similar criteria to categorize patients as having shown clinically significant worsening at post-treatment:

Criteria 1**:** For NF subscales that were clinically elevated at baseline, the follow-up NF score showed at least 50% worsening in the pathological range. The pathological range was defined as the range between the NF subscale specific opening threshold and the baseline score. For example, for a NF scale with an opening threshold of 2, a patient with baseline score of 4 and follow-up score of 5 represents an increase of 50% (1 point increase relative to 2 points of possible improvement between 6 and 4) and would be considered as worsened for that particular NF subscale.

Criteria 2**:** For NF subscales that were clinically elevated at baseline, the follow-up score should be at least 25% higher than the baseline score;

Criteria 3: A patient was categorized as “clinically worsened” if at least 25% of the patient's NF subscales that had been elevated at baseline showed clinical worsening (i.e., met criteria 1 and 2).

### Additional criteria for categorizing patients as clinically improved, clinically worsened, or no change:

If a patient met both the criteria of clinical improvement and worsening as defined in criteria 3, then the patient was categorized as having “clinically improved” (see description below for criteria 3 development).

A patient who did not meet either criteria of clinical improvement or clinical worsening was categorized as “no change”.

# Development of criteria 3:

Given that we had to adapt the Hiller et al. (2011) definition to be applicable to a multidimensional measure, we describe the development of criterion 3 below. To develop a meaningful percentage of subscales needed to demonstrate whether a patient had shown clinically significant improvement or worsening, we used patient and clinician ratings of patient change as a comparator.

### Patient-reported change

As part of the PBH program^1^, patients were asked to provide a rating of self-reported change prior to attending their 6-week follow up visit with the behavioral health integrated clinician. Patients were asked to respond to the question “Compared to when I was first referred to my digital therapeutic treatment, I'm currently feeling” using the following Likert scale:

1. Very much worse since the initiation of treatment
2. Much worse
3. Minimally worse
4. No change from the initiation of treatment
5. Minimally improved
6. Much improved
7. Very much improved since the initiation of treatment

Based on these responses, patients were categorized as “self-report worsened” if they selected any of the following ratings: Very much worse since the initiation of treatment (1), much worse (2) or minimally worse (3). A patient was categorized as “self-report improved” if they rated themselves as: Very much improved since the initiation of treatment (7), much improved (6), or minimally improved (5). Patients who responded “no change from the initiation of treatment” (4) were categorized as “self-report no change”. One hundred sixty-five patients referred to PBH between 2022-12-01 to 2023-07-31 (study timeframe) completed a self-report rating prior to their follow up visit. Table 2 includes the N and percent of patients that were categorized as self-report improved, self-report no change, and self-report worsened.

**Table 2:** Patient self-reported rating of improvement, no change or worsened

| Patient rating | N of Patients (Percentage) |
| --- | --- |
| Self-reported Improved | 122 (73.9%) |
| Self-reported No change | 37 (22.4%) |
| Self-reported Worsened | 6 (3.6%) |

### Clinician-reported change

As part of routine PBH program^2^, the same behavioral health integrated clinician that conducted the initial assessment and referred patients to PBH met with the patient for the 6-week follow up visit. After the visit, the behavioral health integrated clinician was asked to rate the patient’s level of improvement compared to baseline by answering the question “Compared to the patient's condition at referral to the digital therapeutic treatment, this patient's current condition is:” using the Likert scale below:

1. Very much worse since the initiation of treatment
2. Much worse
3. Minimally worse
4. No change from the initiation of treatment
5. Minimally improved
6. Much improved
7. Very much improved since the initiation of treatment

Based on the provider ratings, patients were categorized as “clinician-report worsened” if the clinicians selected any of the following ratings: Very much worse since the initiation of treatment (1), much worse (2) or minimally worse (3). A patient was categorized as “clinician-report improved” if clinicians rated the patients as: Very much improved since the initiation of treatment (7), much improved (6), or minimally improved (5). Clinicians who rated patients as “no change from the initiation of treatment” (4) were categorized as “clinician-report no change”. Clinician reported change data were collected for 245 patients during the study timeframe at the follow-up visit. Table 3 includes the N and percent of patients that were categorized as clinician-report improved, no change or worsened.

**Table 3:** Clinician rating of improvement, no change or worsened

| Clinician rating | N of Patients (Percentage) |
| --- | --- |
| Clinician-report improved | 178 (72.7%) |
| Clinician-report No change | 55 (22.4%) |
| Clinician-report Worsened | 12 (4.9%) |

### Criterion 3 development: combining clinician-rating, patient-rating, and NF subscales

Data were collected from 289 patients referred to PBH between 2022-12-01 to 2023-07-31 (study timeframe) who had a valid NF baseline and follow-up NF. For each patient in the dataset, we extracted the NF subscale(s) that were elevated at baseline. For each patient’s elevated NF subscale, we first applied criteria 1 and 2 (Hiller et al., 2011) described above to determine whether the change fell in the improved, no change, or worsening bucket.

#### *Patient-rating of change and NF subscale percentage thresholds*

To select a minimum percentage threshold of NF subscales that needed to meet criteria 1 and 2 for the patient to be categorized as demonstrating clinical change (either improved, or worsened), we tested how a range of percentage thresholds (from 5% to 100%) in the NF subscales that met criteria 1 and 2 were distanced from the patient-ratings of change. To solution around patients who tied and had the same percentage of improved and worsened NF subscales (based on criteria 1 and 2), we tested two approaches: (a) assign the patient into the “worsened” category; or (b) assign the patient into the “improved” category.

For each of the threshold percentages tested (5-100%) and each of the tie approaches evaluated (assigning to worsened or improved), distance from patient rating was calculated as

2(INF−IR)2+(NNF−NR)2+ (WNF−WR)2−−−−−−−−−−−−−−−−−−−−−−−−−−−−−−−−−−−−√*2(INF−IR)2+(NNF−NR)2+ (WNF−WR)2*

(1)

where,

I_NF_ = improved based on NF subscale change (criteria 1 and 2 met) ; N_NF_ = no change based on NF subscale change ; W_NF_ = worsened based on NF subscale change ; I_R_ = improved based on clinician or patient rating ; N_R_ = no change based on clinician or patient rating ; W_R_ = worsened based on clinician or patient rating.

Distance from improvement rate was weighted twice compared with distance from worsened or no-change to be conservative in our estimation of improvement rates.

Results are summarized in Table 4. The distance from patient rating ranged from 2.14 to 85.84. The closest match to patient rating was obtained using (a) a 25% threshold for criteria 3 and (b) when a patient was assigned as “improved” in case of a tie between the percentage of improved and worsened subscales.

**Table 4:** Results of calculating the distance between patient-rated change and various percentage thresholds of NF subscales meeting criteria as improved or worsened (criteria 1 and 2) by tie criterion assignment

|  | Patient-rated change | | | | | | | |
| --- | --- | --- | --- | --- | --- | --- | --- | --- |
|  | Tie criteria: Patients considered worsened | | | | Tie criteria: Patients considered improved | | | |
| Percentage Threshold for NF subscales | Improved  N (%) | No change  N (%) | Worsened  N (%) | Distance from patient rating | Improved  N (%) | No change  N (%) | Worsened  N (%) | Distance from patient rating |
| 5% | 201(70.3%) | 29(10.1%) | 56(19.6%) | 20.81 | 239 (83.6%) | 29  (10.1%) | 18 (6.3%) | 18.62 |
| 10% | 206(72%) | 31(10.8%) | 49(17.1%) | 18.00 | 237 (82.9%) | 31(10.8%) | 18 (6.3%) | 17.43 |
| 15% | 210(73.4%) | 42(14.7%) | 34(11.9%) | 11.34 | 226(79%) | 42(14.7%) | 18 (6.3%) | 10.89 |
| 20% | 206(72%) | 50(17.5%) | 30(10.5%) | 8.88 | 219 (76.6%) | 50(17.5%) | 17 (5.9%) | 6.62 |
| 25% | 198(69.2%) | 64(22.4%) | 24(8.4%) | 8.20 | **208(72.7%)** | **64(22.4%)** | **14 (4.9%)** | **2.14**** |
| 30% | 184(64.3%) | 88(30.8%) | 14(4.9%) | 16.02 | 187(65.4%) | 88(30.8%) | 11 (3.8%) | 14.67 |
| 35% | 166(58%) | 111(38.8%) | 9(3.1%) | 27.84 | 167(58.4%) | 111 (38.8%) | 8 (2.8%) | 27.39 |
| 40% | 160(55.9%) | 119(41.6%) | 7(2.4%) | 31.91 | 161 (56.3%) | 119 (41.6%) | 6 (2.1%) | 31.47 |
| 45% | 146(51%) | 135(47.2%) | 5(1.7%) | 40.83 | 147(51.4%) | 135 (47.2%) | 4 (1.4%) | 40.4 |
| 50% | 143(50%) | 138(48.3%) | 5(1.7%) | 42.62 | 144(50.3%) | 138(48.3%) | 4 (1.4%) | 42.3 |
| 55% | 109(38.1%) | 176(61.5%) | 1(0.3%) | 64.05 | 109(38.1%) | 176(61.5%) | 1 (0.3%) | 64.05 |
| 60% | 94(32.9%) | 191(66.8%) | 1(0.3%) | 73.10 | 94(32.9%) | 191 (66.8%) | 1 (0.3%) | 73.1 |
| 65% | 91(31.8%) | 194(67.8%) | 1(0.3%) | 74.95 | 91(31.8%) | 194(67.8%) | 1 (0.3%) | 74.95 |
| 70% | 73(25.5%) | 212(74.1%) | 1(0.3%) | 85.84 | 73(25.5%) | 212 (74.1%) | 1(0.3%) | 85.84 |
| 75% | 59(20.6%) | 227(79.4%) | NA | NA | 59(20.6%) | 227 (79.4%) | NA | NA |
| 80% | 56(19.6%) | 230(80.4%) | NA | NA | 56(19.6%) | 230(80.4%) | NA | NA |
| 85% | 47(16.4%) | 239(83.6%) | NA | NA | 47(16.4%) | 239 (83.6%) | NA | NA |
| 90% | 40(14%) | 246(86%) | NA | NA | 40 (14%) | 246(86%) | NA | NA |
| 95% | 40(14%) | 246(86%) | NA | NA | 40 (14%) | 246 (86%) | NA | NA |
| 100% | 40(14%) | 246(86%) | NA | NA | 40 (14%) | 246(86%) | NA | NA |

Note: ** indicates the percentage threshold and tie criterion with closest distance to patient rating

NA = no patients categorized by NF approach under the particular threshold

#### *Clinician-rating of change and NF subscale percentage thresholds*

To achieve the same aim as above for selecting a minimum percentage threshold of NF subscales that needed to meet criteria 1 and 2 for the patient to be categorized as a clinically changed (either improved, or worsened), we also evaluated how a range of percentage thresholds (from 5% to 100%) in the NF subscales that met criteria for 1 and 2 were distanced from the clinician-ratings of change. To solution around patients who tied and had the same percentage of NF subscales determined as improved or worsened (based on criteria 1 and 2), we tested two approaches: (a) assign the patient into the “worsened” category ; or (b) assign the patient into the “improved” category.

For each of the threshold percentages and tie criteria, distance from clinician rating was calculated using equation 1.

Results are summarized in Table 5. The distance from clinician rating ranged from 0 to 85.84. The closest match to patient rating was obtained using (a) a 25% threshold for criteria 3 and (b) when a patient was assigned as “improved” in case of a tie between the percentage of improved and worsened subscales.

**Table 5:** Results of calculating the distance between clinician-rated change and various percentage thresholds of NF subscales meeting criteria as improved or worsened (criteria 1 and 2) by tie criterion assignment

|  | Clinician-rated change | | | | | | | |
| --- | --- | --- | --- | --- | --- | --- | --- | --- |
|  | Tie criteria: Patients considered worsened | | | | Tie criteria: Patients considered improved | | | |
| Percentage Threshold for NF subscales | Improved (%) | No change (%) | Worsened (%) | Distance from clinician rating | Improved (%) | No change (%) | Worsened (%) | Distance from clinician rating |
| 5% | 201(70.3%) | 29(10.1%) | 56(19.6%) | 19.47 | 239 (83.6%) | 29  (10.1%) | 18 (6.3%) | 19.77 |
| 10% | 206(72%) | 31(10.8%) | 49(17.1%) | 16.86 | 237 (82.9%) | 31(10.8%) | 18 (6.3%) | 18.56 |
| 15% | 210(73.4%) | 42(14.7%) | 34(11.9%) | 10.45 | 226(79%) | 42(14.7%) | 18 (6.3%) | 11.86 |
| 20% | 206(72%) | 50(17.5%) | 30(10.5%) | 7.51 | 219 (76.6%) | 50(17.5%) | 17 (5.9%) | 7.45 |
| 25% | 198(69.2%) | 64(22.4%) | 24(8.4%) | 6.06 | **208(72.7%)** | **64(22.4%)** | **14 (4.9%)** | **0**** |
| 30% | 184(64.3%) | 88(30.8%) | 14(4.9%) | 14.55 | 187(65.4%) | 88(30.8%) | 11 (3.8%) | 13.35 |
| 35% | 166(58%) | 111(38.8%) | 9(3.1%) | 26.54 | 167(58.4%) | 111 (38.8%) | 8 (2.8%) | 26.12 |
| 40% | 160(55.9%) | 119(41.6%) | 7(2.4%) | 30.65 | 161 (56.3%) | 119 (41.6%) | 6 (2.1%) | 30.24 |
| 45% | 146(51%) | 135(47.2%) | 5(1.7%) | 39.49 | 147(51.4%) | 135 (47.2%) | 4 (1.4%) | 39.17 |
| 50% | 143(50%) | 138(48.3%) | 5(1.7%) | 41.37 | 144(50.3%) | 138(48.3%) | 4 (1.4%) | 41.07 |
| 55% | 109(38.1%) | 176(61.5%) | 1(0.3%) | 62.80 | 109(38.1%) | 176(61.5%) | 1 (0.3%) | 62.80 |
| 60% | 94(32.9%) | 191(66.8%) | 1(0.3%) | 71.84 | 94(32.9%) | 191 (66.8%) | 1 (0.3%) | 71.84 |
| 65% | 91(31.8%) | 194(67.8%) | 1(0.3%) | 73.67 | 91(31.8%) | 194(67.8%) | 1 (0.3%) | 73.67 |
| 70% | 73(25.5%) | 212(74.1%) | 1(0.3%) | 84.56 | 73(25.5%) | 212 (74.1%) | 1(0.3%) | 84.56 |
| 75% | 59(20.6%) | 227(79.4%) | NA | NA | 59(20.6%) | 227 (79.4%) | NA | NA |
| 80% | 56(19.6%) | 230(80.4%) | NA | NA | 56(19.6%) | 230(80.4%) | NA | NA |
| 85% | 47(16.4%) | 239(83.6%) | NA | NA | 47(16.4%) | 239 (83.6%) | NA | NA |
| 90% | 40(14%) | 246(86%) | NA | NA | 40 (14%) | 246(86%) | NA | NA |
| 95% | 40(14%) | 246(86%) | NA | NA | 40 (14%) | 246 (86%) | NA | NA |
| 100% | 40(14%) | 246(86%) | NA | NA | 40 (14%) | 246(86%) | NA | NA |

Note: ** indicates the percentage threshold and tie criterion with closest distance to clinician rating

NA = no patients categorized by NF approach under the particular threshold

#### *Combining clinician-, patient-ratings with NF subscales*

Taken together, the results show that using a threshold of 25% of elevated NF subscales that met criteria 1 and 2 for change closely captured both patient-rated and clinician-rated changes.

Reference

Hiller W, Schindler AC, Lambert MJ. Defining response and remission in psychotherapy research: a comparison of the RCI and the method of percent improvement. Psychother Res. 2012;22(1):1-11. doi:10.1080/10503307.2011.616237

McAleavey AA, Nordberg SS, Moltu C. Initial quantitative development of the Norse Feedback system: a novel clinical feedback system for routine mental healthcare. Qual Life Res. 2021;30(11):3097-3115. doi:10.1007/s11136-021-02825-1

Nordberg SS, McAleavey AA, Moltu C. Continuous quality improvement in measure development: Lessons from building a novel clinical feedback system. Qual Life Res. 2021;30(11):3085-3096. doi:10.1007/s11136-021-02768-7
