# Supplementary material for: Evaluating the Implementation and Clinical Effectiveness of an Innovative Digital First Care Model for Behavioral Health Using the RE-AIM Framework: Quantitative Evaluation
Source: J Med Internet Res. 2024 Oct 30;26:e54528. doi: 10.2196/54528 (PMC11561446; doi:10.2196/54528)
Supplement: Multimedia Appendix 2 [file jmir_v26i1e54528_app2.docx]

Supplementary material 2.

Demographic comparison between patients with a post-PRO and without a post-PRO. A Welch’s t-test was used to investigate differences in age and chi square tests were used for all other demographic categories.

The results show no significant differences in age t(130) = 0.44, p = 0.66; legal sex χ^2^ (1, N = 410) = 0.08, p = 0.78; race (p=0.11); or ethnicity χ^2^ (2, N = 410) = 0.36, p = 0.83 between the two groups.

|  | **Post-PRO group** | **No post- PRO group** |
| --- | --- | --- |
| Total n | 323 | 87 |
| Age, mean (SD) | 40.33 (15.49) | 40.74 (16.14) |
| Legal sex, *n* (%)  Female  Male  Nonbinary | 252 (78.02)  71 (22.18)  0 | 66 (75.9)  21 (24.14)  0 |
| Race, *n* (%)  White  Black or African American  Asian  Biracial  Native American  Multiracial  Pacific Islander  N/A | 234 (72.45)  17 (5.26)  3 (0.93)  3 (0.93)  2 (0.62)  1 (0.31)  0  63 (19.51) | 53 (61.0)  8 (9.20)  2 (2.30)  0  0  0  0  24 (27.50) |
| Ethnicity, *n* (%)  Non-Hispanic or Latino  Hispanic or Latino  N/A | 194 (60.06)  34 (10.53)  95 (29.41) | 50 (57.47)  11 (12.64)  26 (29. 89) |
